# Fat mass and obesity–associated protein promotes liver steatosis by targeting PPARα

**DOI:** 10.1186/s12944-022-01640-y

**Published:** 2022-03-13

**Authors:** Xiaohui Wei, Jielei Zhang, Min Tang, Xuejiao Wang, Nengguang Fan, Yongde Peng

**Affiliations:** 1 Department of Endocrinology and Metabolism, Shanghai General Hospital, Shanghai Jiao Tong University School of Medicine, 100 Haining Road, 200080 Shanghai, China; 2 Department of Endocrinology, The First Affiliated Hospital of Zhengzhou University, 450052 Zhengzhou, China

**Keywords:** Nonalcoholic fatty liver disease, Fat and obesity associated protein, Peroxisome proliferator-activated receptor α

## Abstract

**Background:**

Nonalcoholic fatty liver disease (NAFLD) is the most common chronic liver disease worldwide. The fat mass and obesity–associated protein (FTO) has been shown to be involved in obesity; however, its role in NAFLD and the underlying molecular mechanisms remain largely unknown.

**Methods:**

FTO expression was first examined in the livers of patients with NAFLD and animal and cellular models of NAFLD by real-time PCR and Western blotting. Next, its role in lipid accumulation in hepatocytes was assessed both *in vitro* and *in vivo* via gene overexpression and knockdown studies.

**Results:**

FTO expression was obviously elevated in the livers of mice and humans with hepatic steatosis, probably due to its decreased ubiquitination. FTO overexpression in HepG2 cells induced triglyceride accumulation, whereas FTO knockdown exerted an opposing effect. Consistent with the findings of *in vitro* studies, adeno-associated viruses 8 (AAV8)-mediated FTO overexpression in the liver promoted hepatic steatosis in C57BL/6J mice. Mechanistically, FTO inhibited the mRNA of peroxisome proliferator-activated receptor α (PPARα) in hepatocytes. Activation of PPARα by its agonist GW7647 reversed lipid accumulation in hepatocytes induced by FTO overexpression.

**Conclusions:**

Overall, FTO expression is increased in NAFLD, and it promotes hepatic steatosis by targeting PPARα.

**Supplementary Information:**

The online version contains supplementary material available at 10.1186/s12944-022-01640-y.

## Background

Nonalcoholic fatty liver disease (NAFLD), characterized by lipid deposition in the liver, has become the most prevalent liver disease affecting approximately 25% individuals worldwide. It encompasses multiple liver pathologies, including simple steatosis, steatohepatitis, liver fibrosis and cirrhosis. In addition, NAFLD is closely related to a series of metabolic disorders, including insulin resistance, type 2 diabetes and cardiovascular diseases [1, 2]. In the past few years, the mechanism of NAFLD has been intensively studied and a substantial emerging pharmacotherapeutic interventions have been proposed [3, 4]. However, to date, no pharmacotherapies for the treatment of NAFLD is approved, partially due to an unclear understanding of its mechanisms [5–7]. Thus, further research is deserved to investigate key players in the pathogenesis of NAFLD and for the development of new therapeutic targets.

N6-methyladenosine (m6A) is the most common modification of mRNA. It is implicated in a broad variety of biological functions, including triglyceride synthesis and energy metabolism [8–10]. The m6A modification of RNA is reversibly regulated by methyltransferases and demethylases [9]. Fat mass and obesity–associated protein (FTO) is the first identified demethylase of m6A modification, and is widely expressed throughout the body including the nervous system, fat, and liver [11–13]. FTO decreases the m6A modification of mRNA and plays an essential role in determining RNA splicing, translation, and degradation [14, 15]. FTO overexpression in mice increases fat mass, whereas its deficiency protects mice against obesity, implying a key role of FTO in obesity [15, 16]. Furthermore, FTO promotes adipogenesis via its m6A demethylation activity [13].

Recently, the role of FTO in lipogenesis in hepatocytes and NAFLD has also been investigated. In the livers of patients with NAFLD and animal models of NAFLD, the expression of FTO is obviously upregulated [17, 18]. Forced FTO expression contributes to a decrease of the m6A level, induces the expression of lipogenic genes, and increases triglyceride accumulation in hepatocytes [19]. However, a mutant FTO deficiency in demethylase activity fails to exert these functions [19]. Similarly, in another study, FTO was found to promote the maturation of the sterol regulatory element-binding protein-1c (SREBP1c) and enhance the transcription of the cell death-inducing DFFA-like effector C (CIDEC) in HepG2 cells [20]. Taken together, FTO appears to be a key player in fat accumulation in hepatocytes. However, the targets of FTO in hepatocytes and its role in NAFLD *in vivo* remain largely unknown.

In the current study, it was found that FTO protein expression was increased in the livers of patients and mice with NAFLD, and the increase in FTO protein may be related to its decreased ubiquitination. FTO contributed to triglyceride accumulation both in hepatocytes *in vitro* and the livers in mice *in vivo*. Mechanistically, FTO expression decreased the mRNA level of peroxisome proliferator-activated receptor α (PPARα). Activation of PPARα reversed the lipid accumulation induced by FTO, indicating that PPARα is a downstream target of FTO.

## Methods

### Chemicals and reagents

Dulbecco’s modified Eagle’s medium (DMEM) and fetal bovine serum (FBS) were obtained from Gibco (Carlsbad, CA, USA). Oil Red O, palmitic acid, low fatty acid bovine serum albumin (BSA), cycloheximide (CHX), GW7647, and MG132, as well as the anti-FLAG M2 affinity gel and antibodies against FLAG, hemagglutinin (HA), and GAPDH were obtained from Sigma-Aldrich (St. Louis, MO, USA). The antibody against FTO was from Abcam (Cambridge, MA, USA) and the anti-PPARα antibody was from ProteinTech Group (Rosemont, IL, USA). The secondary antibodies were all from Jackson Laboratories (West Grove, PA, USA).

### Subjects and liver samples

Liver tissues were obtained from eight subjects who visited the surgical department of the Shanghai General Hospital for liver surgeries of non-hepatocellular primary tumors or liver metastasis of colorectal cancer. Subjects with viral hepatitis and excessive intake of ethanol (> 140 g/week for men or > 70 g/week for women) were excluded. The human study was approved by the Institutional Review Board of Shanghai General Hospital affiliated to Shanghai Jiao Tong University School of Medicine (2020SQ018, 2020). It was performed in accordance with the principle of the Helsinki Declaration II, and written informed consent was obtained from all subjects.

### Animals

Male C57BL/6J mice were obtained from the Model Animal Research Center of Nanjing University (Nanjing, China). The mice were maintained in a pathogen-free facility and allowed free access to diet and water. The procedures were approved by the Committee on the Ethics of Animal Experiments of Shanghai General Hospital affiliated to Shanghai Jiao Tong University School of Medicine (2020SHENG018, 2020).

The NAFLD mouse model was induced by feeding 8-week-old male mice (*n* = 6) with a high-fat diet (HFD) (D12492; Research Diets, New Brunswick, NJ, USA), whereas six control mice were accessed to a standard chow diet (P1300F; SLAC, Shanghai, China). After 16 weeks, about 500 mg livers were obtained and stored at − 80 °C until use.

To overexpress FTO in livers, an adeno-associated virus 8-expressing FTO (AAV8-FTO) vector and its control (an empty vector) were constructed by Jiman (Shanghai, China). Male mice, aged 8 weeks, received a single tail vein injection of 1 × 10^11^ genome copies of the AAV8 or control vector, and were then fed the HFD for 8 weeks. Body weights were recorded weekly.

### Immunohistochemistry and Oil Red O staining

Human liver tissues were fixed in formalin and then embedded in paraffin. Immunohistochemistry was performed on liver Sect. (5 μm) according to standard protocols. Briefly, liver sections were incubated with blocking solution for 1 h at room temperature. Primary antibody against FTO (1:200) was then added and kept at 4 °C overnight. After washing in PBS, slices were incubated with the secondary antibody (1:500) for 1 h. Liver tissues of mice were frozen and sectioned into 12 μm slices, and Oil Red O staining was performed.

### Cell culture and treatments

HepG2 cells were cultured in DMEM supplemented with 10% FBS in a 5% CO_2_ humidified atmosphere at 37°C. For FTO overexpression and knockdown, the lentivirus-FTO vector and its short hairpin (sh)RNA vector were constructed by Heyuan (Shanghai, China). These vectors were transfected into HepG2 cells to establish stable FTO-expressing or FTO-knockdown cell lines. The shRNA target sequence of FTO was: 5’- TCACCAAGGAGACTGCTATTT-3’. Before treatment, hepatocytes were first serum starved for 8 h. Next, palmitic acid (0.5 mM) and 2% BSA were added to the culture medium for 24 h. The HepG2 cells were then fixed with 4% paraformaldehyde and stained with Oil Red O solution (0.2 mg/mL). Relative stained area was quantified by ImageJ software. In addition, the triglyceride quantification colorimetric/fluorometric Kit (BioVision, K622 Milpitas, CA, USA) was applied to quantify the cell triglyceride content following the manufacturer’s protocol.

To determine whether the FTO protein was degraded by proteasomes, HepG2 cells were subjected to the protein synthesis inhibitor CHX (10 µg/mL), with or without the proteasome inhibitor MG132 (10 µM) for 12 h.

### RNA preparation, real-time PCR and RNA sequencing

Total RNA of liver tissue or cells was extracted by the TRIzol reagent (Invitrogen, Carlsbad, CA, USA), and 1 µg of RNA was reverse-transcribed into cDNA by the PrimeScript RT reagent kit (Takara, Otsu, Japan). The qRT-PCR was then performed in triplicate using the SYBR Green qPCR Mix reagent (Toyobo, Osaka, Japan) on an ABI 7500 Real-Time PCR System (Perkin-Elmer Applied Biosystems, Warrington, UK). The primers are listed in Table 1. Relative expression of genes was normalized to GAPDH using the 2 − ΔΔCt method. The RNA transcriptome sequencing analysis was performed by Lianchuan Biotechnology (Hangzhou, China) using the Illumina HiSeq 2500 platform.

**Table 1:** Primers used in real-time PCR

Gene name	Gene ID	Species	Primer Sequence (Forward: 5’-3’)	Primer Sequence (Reverse: 5’-3’)
FTO	79,068	Homo sapiens	ACTTGGCTCCCTTATCTGACC	TGTGCAGTGTGAGAAAGGCTT
PPARα	5465	Homo sapiens	TTCGCAATCCATCGGCGAG	CCACAGGATAAGTCACCGAGG
GAPDH	26,330	Homo sapiens	TGGATTTGGACGCATTGGTC	TTTGCACTGGTACGTGTTGAT
FTO	26,383	Mus musculus	TTCATGCTGGATGACCTCAATG	GCCAACTGACAGCGTTCTAAG
GAPDH	14,433	Mus musculus	AGGTCGGTGTGAACGGATTTG	TGTAGACCATGTAGTTGAGGTCA

### Western blotting analyses

Liver tissues and cell samples were collected with sodium dodecyl sulfate (SDS) lysis buffer. Protein lysates were subjected to 10% SDS-polyacrylamide gel electrophoresis and transferred to PVDF membranes (EMD Millipore, Bedford, MA, USA). After incubation with the indicated primary antibodies overnight at 4 °C (all primary antibodies were diluted at 1:1,000), the membranes were incubated with horseradish peroxidase-conjugated secondary antibodies (1:2000 dilution) for 1 h at room temperature. The target proteins were visualized using Super Signal West Pico Chemiluminescent Substrate (Thermo Scientific, Rockford, IL, USA). GAPDH was used as the internal control. Protein bands were quantified via densitometric analyses using the Image J software.

### Protein immunoprecipitation and ubiquitination assays

The HA-ubiquitin plasmid was transiently transfected into the stable cell line overexpressing FLAG-FTO with Lipofectamine 3000 (Invitrogen). Ubiquitination of FTO was induced by treating HepG2 cells with 10 µM MG132. Briefly, cells were lysed in 1% SDS and diluted in ice-cold cell lysis buffer. Cell lysates were sonicated and centrifuged at 9000 x g for 20 min at 4 °C, and then incubated with the anti-FLAG M2 Affinity Gel overnight at 4 °C. After washes, the immunoprecipitated proteins were resolved via SDS-polyacrylamide gel electrophoresis and subjected to immunoblotting with specific antibodies against HA or FTO.

### Statistical analyses

Statistical analyses were conducted using the Prism 7.0 software (GraphPad Software, San Diego, CA, USA). Data are presented as mean ± SD. Cellular experiments were repeated at least three times. A two-tailed unpaired Student’s t-test was performed for comparisons between two groups. The animal data were analyzed using non-parametric statistical tests. *P* < 0.05 was considered statistically significant.

## Results

### FTO expression is increased in fatty liver

**Fig. 1:**
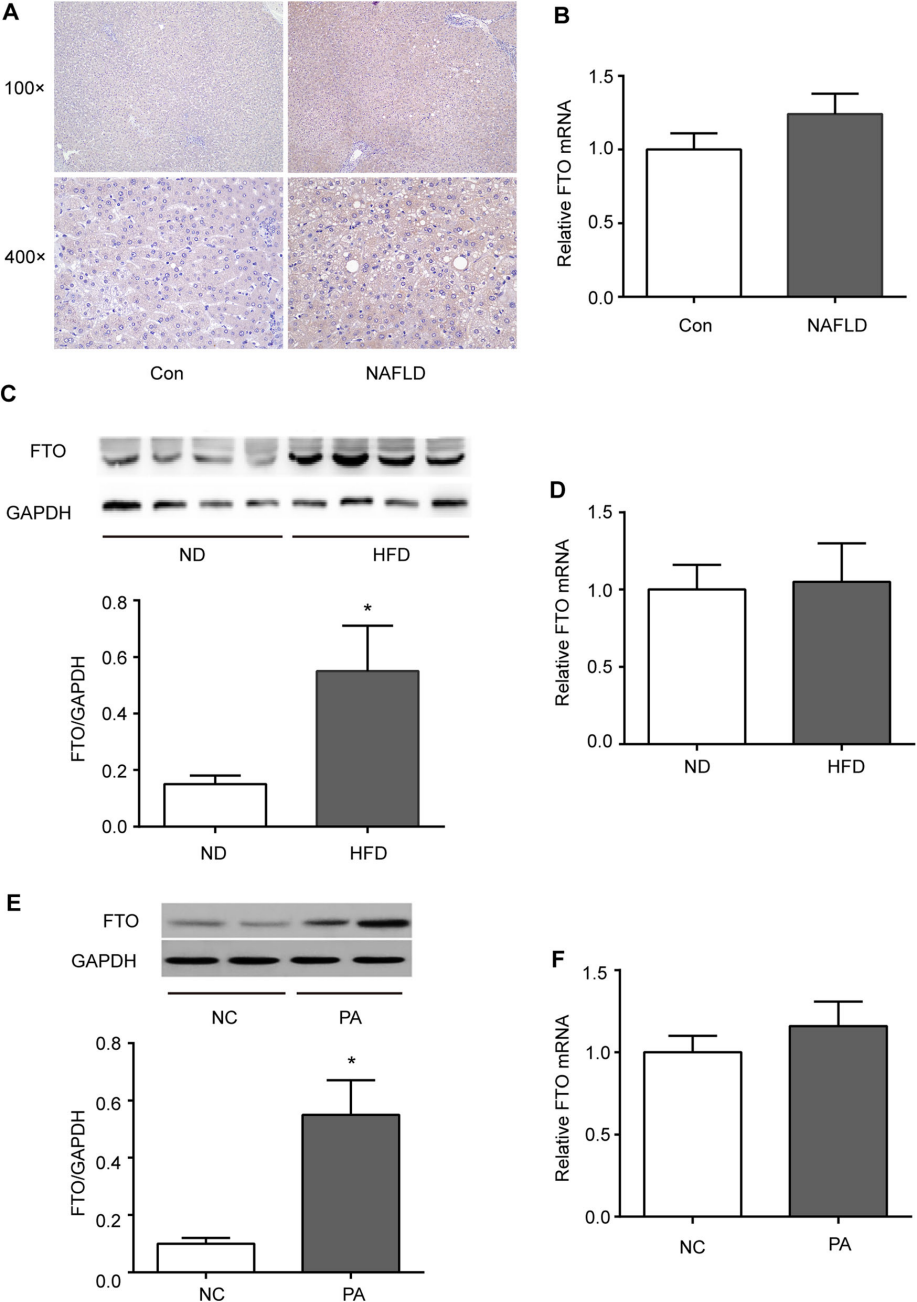
Upregulation of FTO in nonalcoholic fatty liver disease (NAFLD). **A, B** Immunohistochemical and quantitative real-time polymerase chain reaction (qRT-PCR) analyses of FTO protein and mRNA expression in the livers from patients with and without NAFLD (*n* = 4 per group). **C, D** Western blotting and qRT-PCR analyses of the FTO protein and mRNA expression in the livers of mice fed a high-fat diet (HFD) or normal diet (ND) for 16 weeks (*n* = 6 mice per group). Band intensities were quantified via densitometry. Relative expression was normalized to GAPDH and is expressed as the fold-change relative to the controls. **E, F** Western blotting and qRT-PCR analyses of the FTO protein and mRNA expression in HepG2 cells treated with palmitic acid (PA) (0.5 mM) for 24 h. Band intensities were quantified via densitometry. *P < 0.05 vs. control group

To explore the role of FTO in fatty liver, its expression was first determined in the livers of human subjects with or without NAFLD. Immunohistochemistry revealed that FTO protein levels were expressed in hepatocytes of liver and tended to increase in NAFLD (Fig. 1 A). FTO mRNA was comparable between the livers from patients with and without NAFLD (Fig. 1B).

FTO expression was examined in a mouse model of hepatic steatosis induced by feeding mice with HFD for 16 weeks. Consistent with our observation in humans, FTO protein level was obviously higher in the livers of HFD-fed mice when compared with those in the controls (Fig. 1 C). *In vitro*, FTO protein was also significantly increased in HepG2 cells after challenged with palmitic acid (Fig. 1E). In contrast, FTO mRNA was not distinctly changed in fatty livers of mice or in palmitic acid–treated HepG2 cells (Fig. 1D, F). This suggests that the FTO protein in fatty liver may be regulated at the post-transcriptional level.

### Palmitic acid decreases ubiquitination of FTO in hepatocytes

**Fig. 2:**
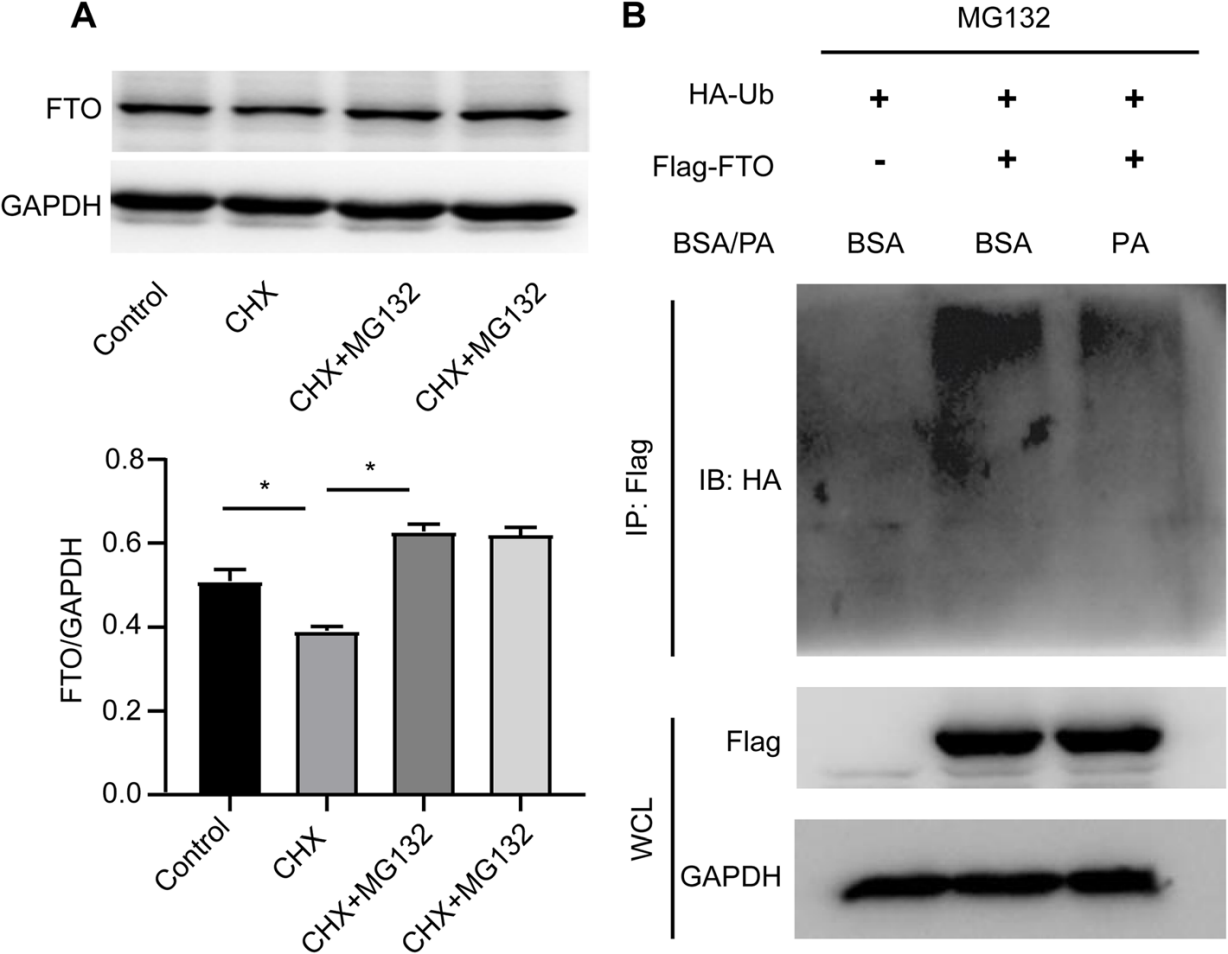
Palmitic acid (PA) decreases the ubiquitination of FTO in hepatocytes. **A** Western blotting analysis of FTO protein expression in HepG2 cells treated with the protein synthesis inhibitor cycloheximide (CHX, 10 µg/mL), and/or the proteasome inhibitor MG132 (10 µM) for 24 h. Band intensities were quantified via densitometry. **B** HepG2 cells overexpressing FLAG-FTO or negative control vectors were transfected with HA-ubiquitin and treated with PA (0.5 mM) for 12 h. The ubiquitination of FTO was assessed via immunoprecipitation and western blotting analyses

Previous study has shown that FTO is modified by ubiquitination and degraded in proteasomes in HeLa cells [21]. Considering the discrepancy in changes of FTO protein and mRNA in NAFLD, it was speculated that FTO may be dysregulated by the ubiquitin-proteasome system in fatty liver. To test this hypothesis, HepG2 cells were subjected to the protein synthesis inhibitor CHX with or without the proteasome inhibitor MG132. Consistently, it was observed that MG132 could prevent the decrease of FTO protein level in HepG2 cells treated with CHX (Fig. 2 A). This indicated that FTO was degraded by proteasomes in hepatocytes. To confirm whether FTO protein upregulation in fatty liver was related to its ubiquitination, the ubiquitination level of FTO was evaluated in hepatocytes exposed to palmitic acid as an *in vitro* model of fatty liver. As shown in Fig. 2B, the ubiquitination of FTO was markedly decreased by exposure to palmitic acid. Thus, changes in ubiquitination may be responsible for the increase in FTO protein expression in NAFLD.

### FTO promotes triglyceride deposition in hepatocytes

**Fig. 3:**
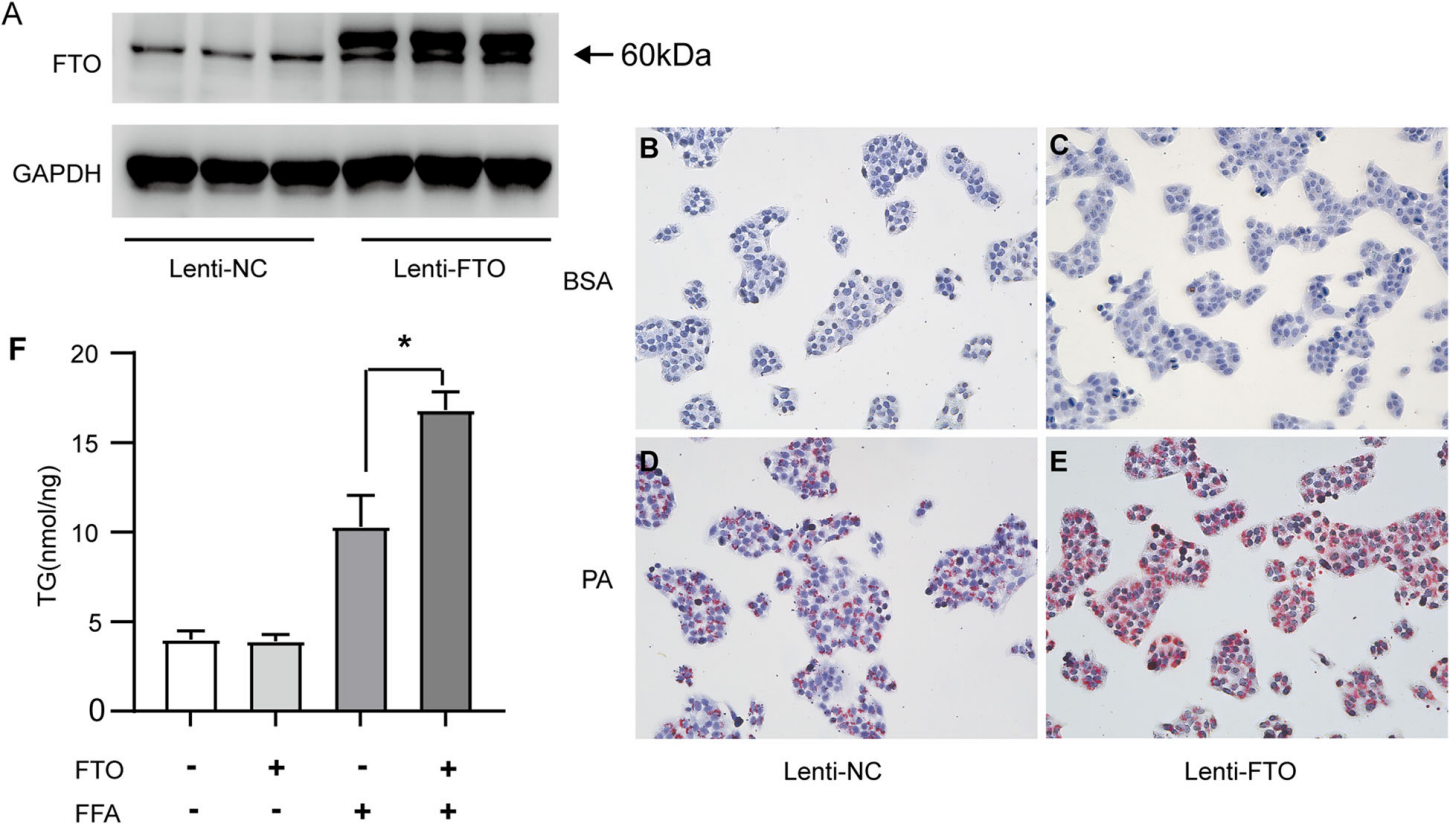
FTO overexpression induces lipid accumulation in hepatocytes. **A** Western blotting analysis of the FTO protein level in HepG2 cells transfected with the FLAG-FTO lentiviral (lenti-FTO) or negative control (lenti-NC) vector. **B-E** Oil Red O staining of HepG2 cells overexpressing FLAG-tagged FTO or the empty vector were treated with palmitic acid (PA) (0.5 mM) or 2% bovine serum albumin (BSA) for 24 h (200x magnification). **F** Cellular contents of triglycerides (TG) in FTO-overexpressing and control HepG2 cells stimulated with 2% BSA or PA (0.5 mM) for 24 h (*n* = 3 independent experiments). *P < 0.05 vs. control group

To probe the role of FTO on triglyceride deposition in hepatocytes, HepG2 cells were first infected with a lentivirus vector-mediated FTO plasmid (Lenti-FTO) and a stable cell line overexpressing FTO was obtained (Fig. 3 A). As revealed by Oil Red O staining, lipid deposition was increased in FTO-overexpressing cells compared with that in control cells after treatment with palmitic acid (Fig. 3B-E). In addition, hepatocytes with FTO overexpression exhibited a significantly higher level of triglycerides than control cells (Fig. 3 F).

**Fig. 4:**
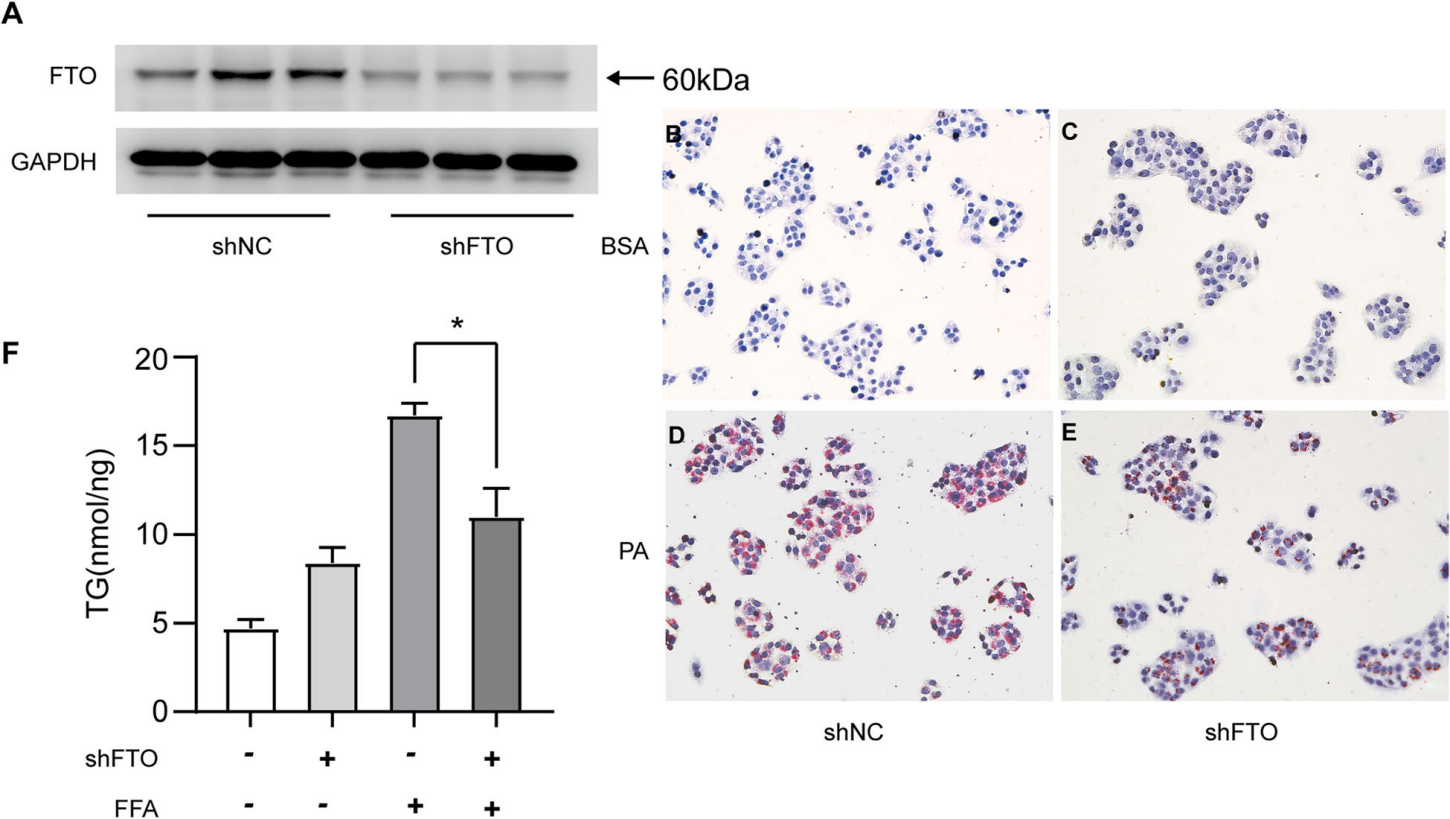
Knockdown of FTO inhibits lipid accumulation in hepatocytes. **A** Western blotting analysis of the FTO protein level in HepG2 cells transfected with the FTO shRNA lentiviral (shFTO) or negative control (shNC) vector. **B-E** Oil Red O staining of HepG2 cells transfected with shFTO or shNC after treatment with palmitic acid (PA) (0.5 mM) or 2% bovine serum albumin (BSA) for 24 h (200x magnification). **F** Cellular contents of triglycerides (TG) in FTO knockdown and control HepG2 cells stimulated with 2% BSA and PA (0.5 mM) for 24 h (*n* = 3 independent experiments). *P < 0.05 vs. control group

To confirm the role of FTO in lipid deposition in hepatocytes, a stable cell line with FTO knockdown was generated by infecting HepG2 cells with a lentivirus vector-mediated FTO shRNA plasmid. In contrast to FTO overexpression, knockdown of FTO markedly inhibited lipid accumulation in hepatocytes, as assessed by Oil Red O staining and triglyceride measurements (Fig. 4).

### FTO promotes hepatic steatosis in mice

**Fig. 5:**
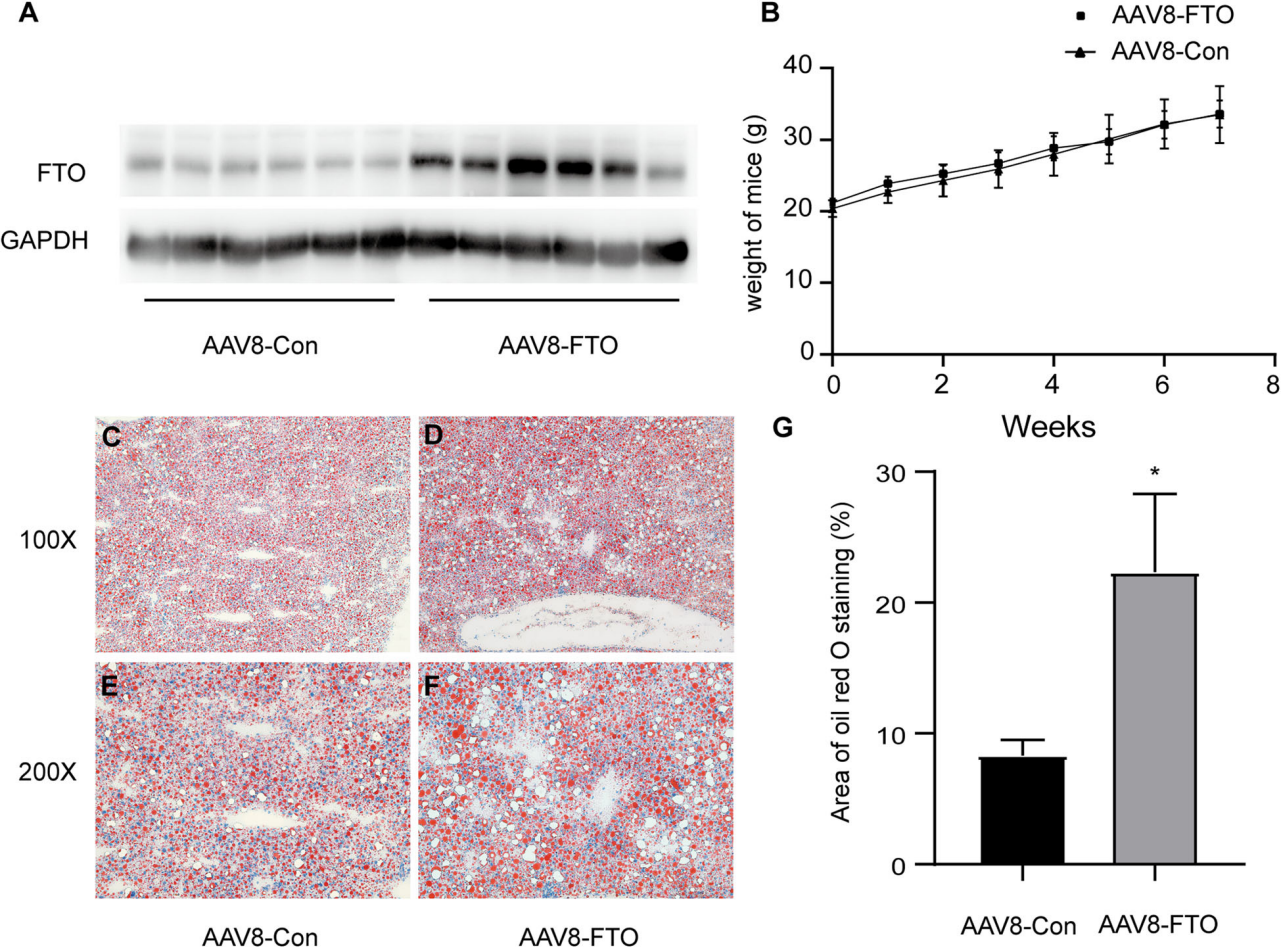
FTO promotes hepatic steatosis in mice. **A, B** C57BL/6J male mice were administered the AAV8-FTO (AAV8-FTO) or control (AAV8-Con) vector by tail vein injection, then fed a high-fat diet (HFD) for 8 weeks. Body weights were recorded at the indicated time points (*n* = 6 mice per group). **C-F** Representative images of Oil Red O staining (200x magnification) in the liver tissues of FTO-overexpressing and control mice fed an HFD for 8 weeks (*n* = 6 mice in each group). **G** Quantification of Oil Red O staining area in hepatocytes by the ImageJ software

To address whether it promotes hepatic steatosis *in vivo*, FTO was overexpressed in the liver of mice by AAV8-FTO via tail vein injection. The mice were then fed an HFD for eight weeks, and hepatic steatosis was assessed via Oil Red O staining. As shown in Fig. 5 A and B, FTO overexpression had little effect on the body weights of the mice. However, the livers with FTO overexpression exhibited more prominent steatosis when compared with those of the controls (Fig. 5 C-G).

### FTO inhibits the expression of PPARα

**Fig. 6:**
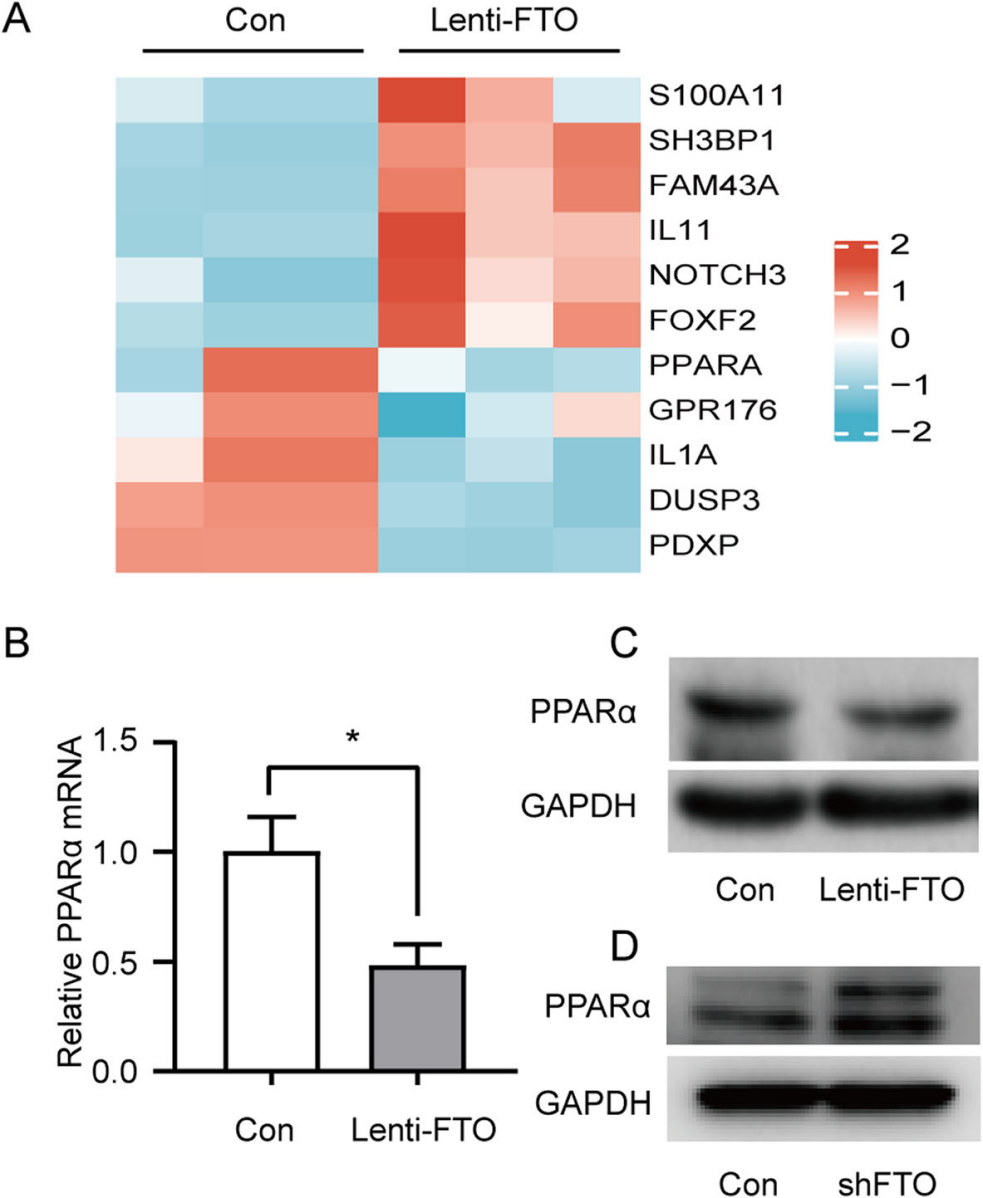
FTO inhibits the expression of PPARα. **A** Heat map of differentially expressed mRNAs between FTO-overexpressing and control HepG2 cells via high-throughput sequencing (*n* = 3 per group). **B, C** Quantitative real-time polymerase chain reaction and western blotting analyses of PPARα mRNA and protein expression in FTO-overexpressing and control HepG2 cells. **D** Western blotting analysis of PPARα protein expression in FTO-knockdown and control HepG2 cells (*n* = 3 independent experiments)

FTO is a main RNA demethylase, and its demethylation function is important in the lipogenesis of hepatocytes. However, the downstream targets of FTO are not yet well-understood. To identify the downstream targets of FTO in hepatocytes, RNA sequencing was performed on samples from FTO-overexpressing and control HepG2 cells. Genes involved in multiple metabolic pathways were identified, including PPARα (Fig. 6 A). The inhibitory role of FTO on PPARα mRNA and protein expression was then determined in HepG2 cells. As shown in Fig. 6B and C, FTO overexpression in HepG2 cells reduced both PPARα mRNA and protein levels. Consistent with this finding, FTO knockdown by shFTO increased PPARα protein levels (Fig. 6D).

### FTO induces lipid accumulation in hepatocytes by targeting PPARα

**Fig. 7:**
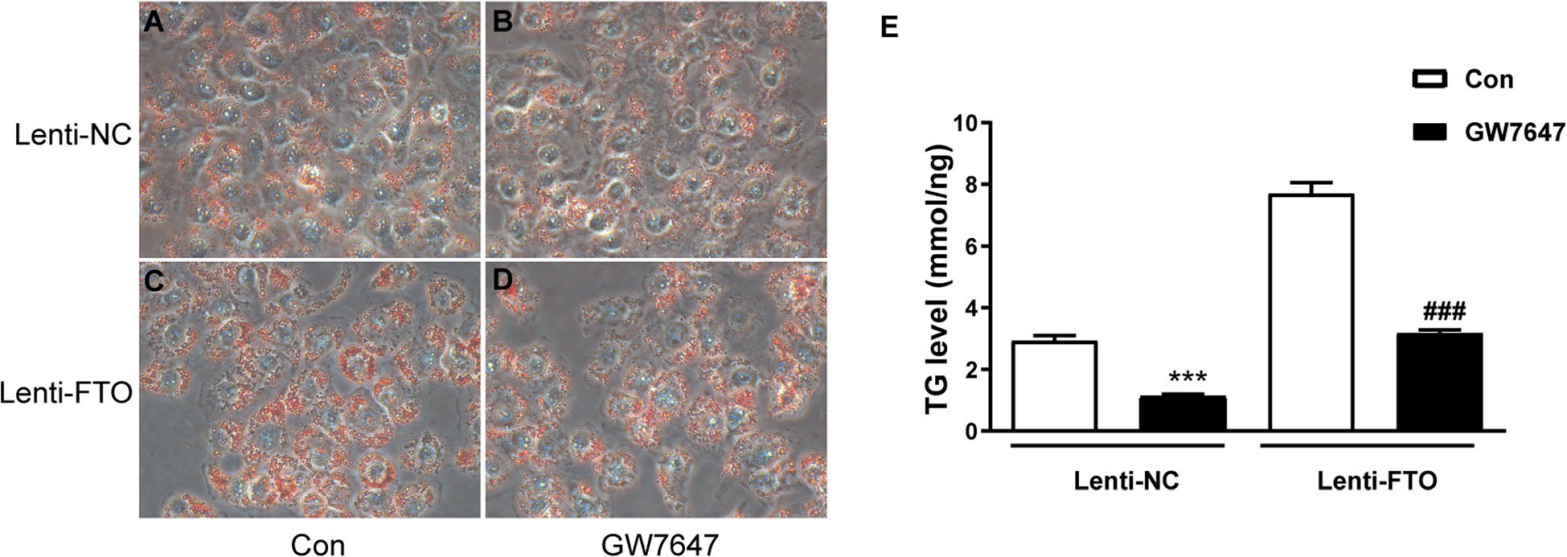
FTO induces lipid accumulation in hepatocytes by targeting PPARα. **A-D** FTO-overexpressing (Lenti-FTO) and control HepG2 cells (Lenti-NC) were treated with palmitic acid (PA) (0.5 mM) for 24 h without (**A, C**) or with (**B, D**) challenge with the PPARα agonist, GW7647 (10 µM). Oil Red O staining was performed and photographed at 200x magnification (*n* = 3 independent experiments)

PPARα is a key regulator of fatty acid oxidation, and downregulation of PPARα contributes to hepatic steatosis and NAFLD [22]. To test whether FTO promotes lipid accumulation by targeting PPARα, FTO-overexpressing HepG2 cells were treated with the PPARα agonist GW7647. As expected, GW7647 reversed the triglyceride deposition in HepG2 cells induced by FTO overexpression, as evidenced by Oil Red O staining as well as triglyceride measurements (Fig. 7).

## Discussion

FTO, the first identified m6A demethylase, promotes adipogenesis and obesity [14–16]. In addition, it has been found to induce triglyceride deposition in hepatocytes [19, 20]. However, its role in NAFLD is largely unknown. In the current study, FTO was shown to promote hepatic steatosis *in vivo*. Moreover, FTO inhibited the expression of PPARα, which may mediate its role in hepatic steatosis.

In previous studies, FTO expression was found to be increased in the livers of patients with NAFLD and mice fed an HFD for 6–12 weeks [17, 18, 23]. However, there are inconsistencies regarding the expression of FTO in NAFLD. FTO mRNA level was reduced in genetically obese mice [24]. In contrast, its expression was not changed both in mRNA and protein level in the livers of mice fed an HFD for 17 weeks [25]. In the present investigation, FTO protein level was increased in the livers of mice fed an HFD for 16 weeks, whereas its mRNA level was not significantly changed. Differences in species and/or the duration of HFD feeding may be responsible for these inconsistent findings. Because FTO can be modified by ubiquitination and subsequently degraded by proteosomes, it was speculated that dysregulated ubiquitination of the FTO protein was responsible for the discrepancy in changes of FTO protein and its mRNA levels in NAFLD. In support of our speculation, FTO ubiquitination was inhibited in hepatocytes challenged with palmitic acid, which may lead to decreased degradation of FTO and the upregulation of its expression in NAFLD.

The involvement of FTO in lipid metabolism of hepatocytes has been investigated *in vitro* previously. FTO contributes to lipid accumulation in HepG2 cells [19, 20]. Consistently, present study shows that FTO overexpression induced triglyceride accumulation in hepatocytes, whereas FTO knockdown inhibited this accumulation. However, whether FTO takes part in hepatic steatosis *in vivo* has not yet been investigated. In the present study, FTO was overexpressed specifically in the livers of mice by AAV8 and it promoted hepatic steatosis, consistent with the *in vitro* studies. Meanwhile, hepatic overexpression of FTO did not affect the body weights of mice, implying a direct role for FTO in liver steatosis. The current study provides further evidence that FTO exerts a key role in the pathogenesis of NAFLD.

Another question was how FTO promoted lipid accumulation in hepatocytes. Previous studies proposed that SREBP1c is a downstream molecular target of FTO [20]. In addition, FTO was found to decrease the mitochondria content, induce the expression of genes implicated in lipogenesis (FASN, SCD1, and MOGAT1), and decrease the levels of genes in lipid transport (MTTP, APOB, and LIPC) [19]. Furthermore, FTO contributes to lipid accumulation in hepatocytes via its demethylase function, as FTO deficiency in demethylase activity leads to loss of these effects [19]. Our study showed that FTO decreased the mRNA level of PPARα. As PPARα is a master regulator implicated in fatty acid oxidation, reduction of its level would contribute to lipid accumulation in hepatocytes. As expected, activation of PPARα reversed the lipid accumulation in hepatocytes induced by FTO overexpression in our study. Altogether, FTO may promote hepatic steatosis via multiple mechanisms, including effects on de novo lipogenesis, lipid transport, and fatty acid oxidation.

Though FTO decreased the expression of PPARα, the underlying mechanism is yet unknown. Since FTO functions as a demethylase of RNA, it deserves further study whether FTO directly decreases the methylation of PPARα mRNA and changes its degradation.

## Study strengths and limitations

The present study showed that FTO was a key player of fatty liver, which is a potential target for the treatment of NAFLD. However, the first limitation is that the liver weight, hepatic triglyceride and liver tests were not measured in FTO overexpression mice. Second, this study did not investigate the effect of deletion of FTO on liver steatosis. Another limitation is that whether FTO directly interacts with and demethylates PPARα is not investigated in the study, and remains to be determined in the future investigation.

## Conclusions

In summary, FTO is upregulated in NAFLD and suppresses the expression of PPARα in hepatocytes, leading to hepatic steatosis. These findings provide insight into the mechanism of NAFLD and suggest that FTO may be a novel target for the treatment of NAFLD.

## Supplementary information

**Additional file 1.**

**Additional file 2.**

**Additional file 3.**

## Data Availability

All data related to this study are available upon request.

## Abbreviations

NAFLD: Nonalcoholic fatty liver disease
FTO: Fat and obesity associated protein
PPARα: Peroxisome proliferator-activated receptorα
FASN: Fatty acid synthase
SCD1: Stearoyl-CoA desaturase 1
MOGAT1: Monoacylglycerol O-acyltransferase 1
MTTP: Microsomal triglyceride transfer protein
APOB: Apolipoprotein B
LIPC: Lipase C
