# Supplementary material for: Fat mass and obesity–associated protein promotes liver steatosis by targeting PPARα
Source: Lipids Health Dis. 2022 Mar 13;21:29. doi: 10.1186/s12944-022-01640-y (PMC8918283; doi:10.1186/s12944-022-01640-y)
Supplement: Supplementary file 1 — Additional file 1. [file 12944_2022_1640_MOESM1_ESM.docx]

**Supplementary Information**

**Figure legend**

Supplementary Figure 1. The effect of FTO on the expression of PPARγ. Quantitative real-time polymerase chain reaction analyses of PPARγ mRNA expression in FTO-overexpressing and control HepG2 cells.

Supplementary Figure 2. The effect of FTO on the expression of SREBP1c. Quantitative real-time polymerase chain reaction analyses of SREBP1c mRNA expression in FTO-overexpressing and control HepG2 cells.

Supplementary Table 1. Comparison of expressed mRNAs between FTO-overexpressing and control HepG2 cells via high-throughput sequencing.

| **gene_name** | **log2FC** | **p_value** | **LVF** | **Con** | **LVF1** | **FVF2** | **LVF3** | **Con1** | **Con2** | **Con3** |
| --- | --- | --- | --- | --- | --- | --- | --- | --- | --- | --- |
| SREBP1c | -0.25 | 0.16 | 20.38 | 24.29 | 16.37 | 19.84 | 21.94 | 21.33 | 21.33 | 27.21 |
| PPARD | -0.04 | 0.91 | 10.20 | 10.54 | 8.27 | 9.98 | 9.35 | 11.07 | 11.07 | 6.48 |
| PPARG | -0.00 | 0.92 | 6.55 | 6.58 | 4.92 | 5.78 | 5.94 | 5.77 | 5.77 | 5.21 |

LVF: lenti-virus mediated FTO overexpression.
