# Supplementary figures and images for: Fat mass and obesity–associated protein promotes liver steatosis by targeting PPARα
Source: Lipids Health Dis. 2022 Mar 13;21:29. doi: 10.1186/s12944-022-01640-y (PMC8918283; doi:10.1186/s12944-022-01640-y)

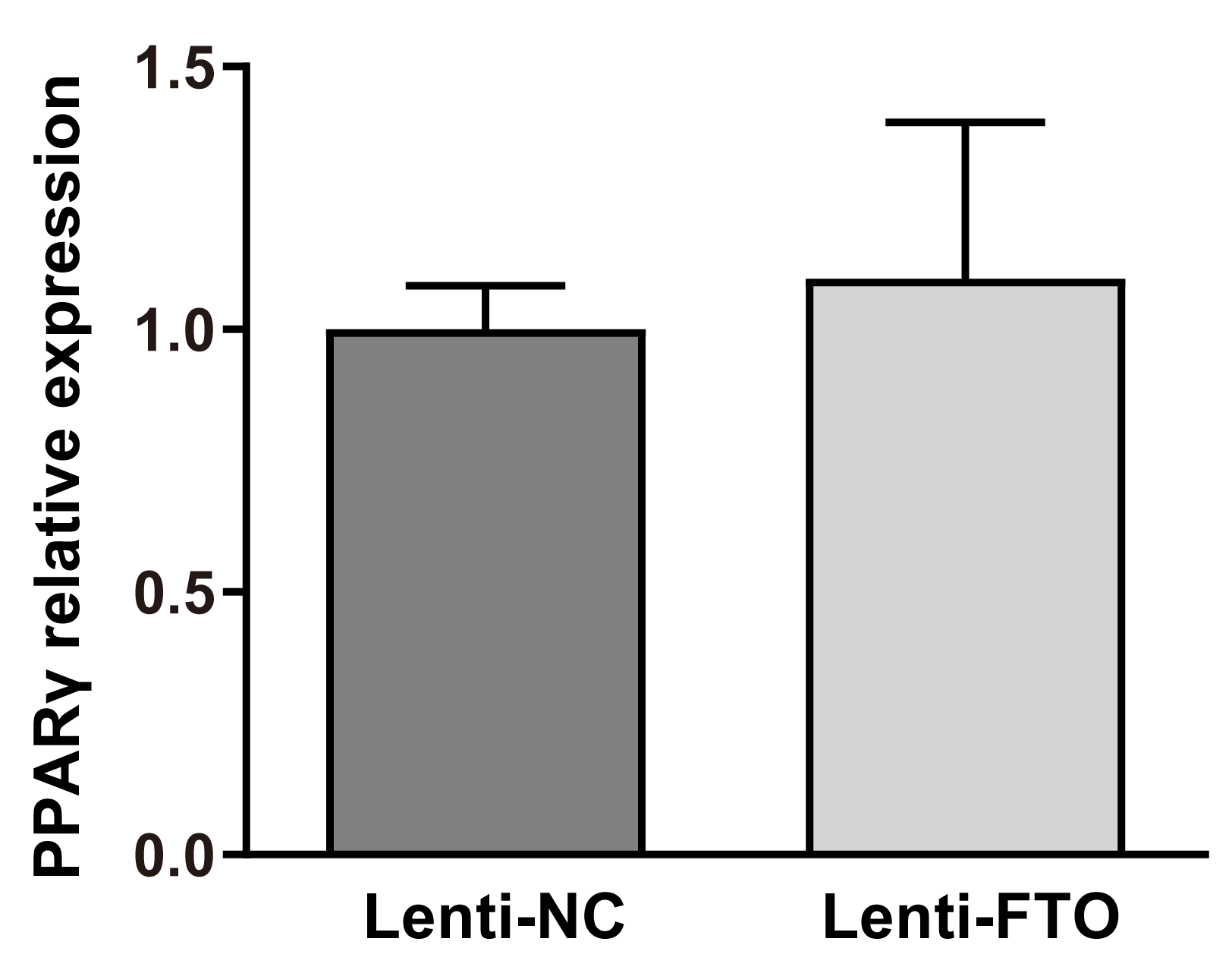

Supplement: Supplementary file 2 — Additional file 2. [file 12944_2022_1640_MOESM2_ESM.tif]

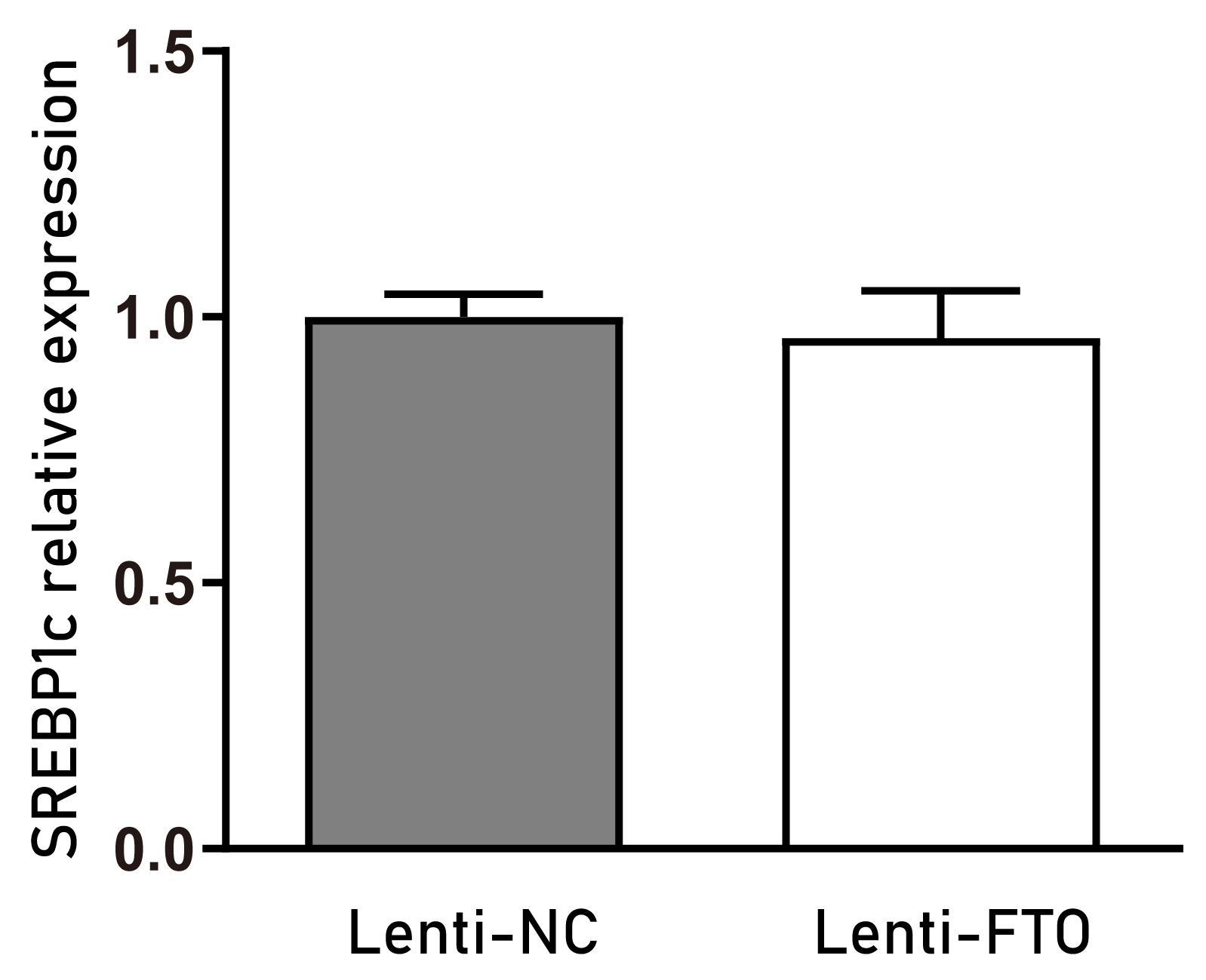

Supplement: Supplementary file 3 — Additional file 3. [file 12944_2022_1640_MOESM3_ESM.tif]
